# Comparison of the Accuracy of a Mounting Fixture for Dental Implants for Implant Position Transfer and Open-Tray Implant Level Impression—An In Vitro Study

**DOI:** 10.3390/dj11090208

**Published:** 2023-08-31

**Authors:** Alexander Becker, Dieter Dirksen, Christoph Runte

**Affiliations:** Department of Prosthetic Dentistry and Biomaterials, University of Muenster, D-48149 Münster, Germany; dirksdi@uni-muenster.de (D.D.); christoph.runte@ukmuenster.de (C.R.)

**Keywords:** implant impression, implant position transfer, prosthetic restoration on implants

## Abstract

The accuracy for the implant position transfer of a mounting fixture and a standardized open-tray implant level impression was compared. Ten aluminum master models with four implant analogs placed in different angulations were fabricated. By performing an open-tray implant level impression stone casts were produced. The master models and stone casts were scanned (comparison group one) using a laboratory scanner. Deviations in the scan body surface were determined in the form of mean (absolute) point distances and (signed) surface distances. The same procedure was performed with a screwed transfer and by fixing the posts of the mounting fixture (comparison group two). The mounting device was applied to each master model and scanned in a fixed and detached state (comparison group three). In a point comparison, the open-tray implant level impression showed mean deviations of 43.6 µm and a mounting fixture of 44.6 µm with no significant differences (*p* < 0.05). There were significant differences between groups two and three. The angulation of the implants had no effect on the accuracy. In a surface comparison, the open-tray implant level impression showed mean deviations of 36.0 µm and a mounting fixture of 2.0 µm (*p* > 0.05). Within the limits of this study, the mounting fixture transferred the implant position with the same accuracy as the open-tray implant level impression with respect to point deviations.

## 1. Introduction

Oral implantology with subsequent prosthetic restorations has been expanding the therapeutic spectrum of surgical–prosthetic dentistry for years. Edentulous jaws, shortened dental arches or interdental gaps can be treated with fixed or removable dentures through implantation with higher comfort and a better fit. For these treatments, immediate implant stability with subsequent full osseointegration is a prerequisite. However, there is a difference in the mobility of natural teeth and dental implants. Periodontally healthy teeth show a physiological mobility between 20 µm and 100 µm [1,2]. In comparison, an implant has a tenfold-reduced mobility of approx. 2 µm [2]. These characteristics require an implant impression as accurate as possible to obtain a stress-free superstructure. Stress in superstructures can cause biological or mechanical complications, such as gaps, peri-implantitis, occlusal inaccuracies or the loosening of the superstructure, the screw or the implant [3,4,5,6].

The accuracy of the implant impression is affected, inter alia, by the implant angulation, impression technique, additional splinting, impression material and cast material [7,8,9,10,11,12,13,14,15,16,17]. Polyether and polyvinyl siloxane materials are most frequently used for implant impressions. Both materials show small inaccuracies in the transfer of the implant impression [18,19]. Due to expansion, the master cast can show altered dimensions compared to the oral situation [20,21]. A digital impression can be an alternative, but it is also affected by inaccuracies such as the experience of the clinician, matching of scan data or volume of the scan [22,23,24,25]. The open and closed implant impression techniques have been the most common and proven procedures over past decades. The open-tray implant level impression appears to have a higher accuracy when used for full-arch impressions, angulated implants or more than four inserted implants [7,8,26,27].

The accuracy of the implant position transfer has been shown to depend on the implant angulation [14,26]. If the implant angulation is only small, the effect seems to not be clinically relevant [28,29,30].

Nt-trading (Karlsruhe, Germany) developed the nt-VAL-Jig in 2018 to achieve a transfer of the implant position of four implants with high accuracy. The aim of applying the nt-VAL-Jig is to control implant positions on the stone cast produced using a conventional impression. A correction of the implant analog position of the stone cast with this device should be possible too. Being able to take an extraoral scan of the nt-VAL-Jig for the calculation of the implant position to achieve a digital workflow is intended in the future.

The aim of this in vitro study was to investigate whether the nt-VAL-Jig transfers implant positions more accurately than the open implant impression. We hypothesized that the nt-VAL-Jig would transfer the implant position with higher accuracy than the open implant impression. As the open nonsplinted impression technique can be regarded as the accepted gold standard in implant impression taking [8], our experimental setup aimed to answer whether the nt-VAL-Jig was capable of detecting deviations between the original implant position on the master model and the plaster-cast-embedded laboratory implant position.

## 2. Materials and Methods

### 2.1. Master Model Construction

**Figure 1:**
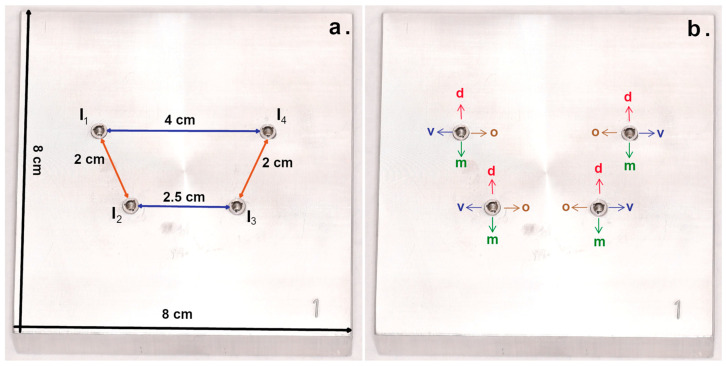
Labeling of the master model ((**a**) dimension and labeling of implants; (**b**) direction designation).

Ten aluminum master models with a length and width of 8 cm and a height of 2 cm were fabricated. Each master model contained four holes drilled exactly for implant analogs for the Camlog implant system (CAM 5.La3.800; nt-trading). The holes were drilled in different angulations in a region corresponding to the mandibular canine teeth and the mandibular molar teeth (Table 1 and Figure 1). The anterior implants were positioned at a distance of 25 mm, the anterior to the posterior ones at a distance of 20 mm, as measured from the center of each analog. The implant analogs were fixed with superglue (Pluline superglue; Pluradent, Offenbach, Germany) and were named as shown in Figure 1.

### 2.2. Impression Tray Design

**Figure 2:**
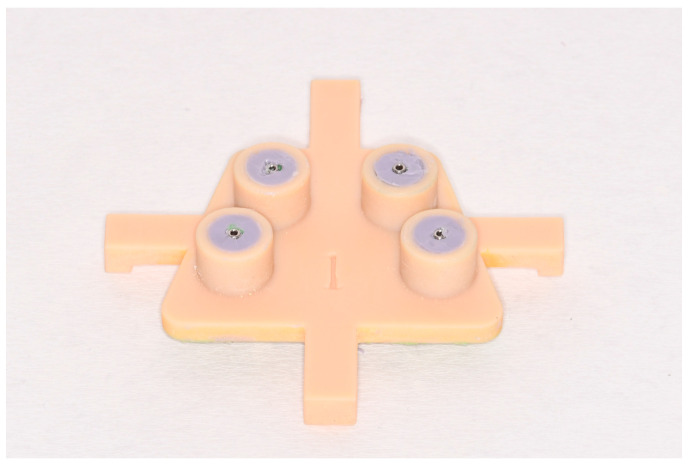
Custom tray with extensions.

To increase the accuracy of the impression and obtain a nearly uniform layer thickness of the impression material around the impression copings, custom trays were fabricated for each master model. For this purpose, the ten master models were digitized with a laboratory scanner and screwed scan bodies (Arctica AutoScan and Dental Teacher 4.0; KaVo, Biberach, Germany, and CAM 9.S3D3.800; nt-trading). The custom impression trays were designed with Geomagic Freeform 2018 (3D Systems; Rock Hill, SC, USA) with tubular sockets at a distance of 5 mm to the implant axis. The height of the created cylinders was 10 mm. To achieve a definite fit of the tray during the impression, four extensions were designed, which encompassed the margins of the master model (Figure 2). With the same software, a mold for casting the impression with plaster was created. The custom trays and mold were printed in resin (Objet MED690; Stratasys, Eden Prairie, MN, USA) with the Objet Eden 260 3D-Printer (Stratasys).

### 2.3. Impression Protocol

The experimental procedure was performed under a constant room temperature of 23° centigrade. All materials were used according to the manufacturer’s instructions. One open-tray implant level impression was created from each master model with the specific custom tray. Therefore, impression copings for the Camlog implant system (CAM TR-00023.8; nt-trading) were hand-tightened onto the implant analogs. Polyvinyl siloxane impression material (Flexitime Heavy Tray and Flexitime Correct Flow; Kulzer, Hanau, Germany) was dispensed using a mixing unit (Dynamix Speed; Kulzer, Hanau, Germany) and a dispensing gun. A stopwatch was used to standardize the time for loading the tray, injecting the impression material around the copings, seating the tray and full setting. After the full setting of the impression material, the copings were unscrewed and the impressions removed from the master cast. Implant analogs for the Camlog implant system (CAM 5.La3.800; nt-trading) were hand-tightened onto the impression copings. Then, each impression rested for 60 min.

### 2.4. Stone Cast Production Protocol

First, the casting mold was insulated with Vaseline (Vaselin Salbe LAW; Abanta Pharma GmbH, Plettenberg, Germany) in order to be able to better demold the created plaster models. Every single impression was placed in the casting mold. Type IV dental stone (Silky-Rock-Yellow; Whip Mix, Louisville, KY, USA) was used according to the manufacturer’s instructions. Each stone cast was then trimmed to a height of 20 mm and rested for seven days to achieve the full setting expansion of the plaster. All laboratory work was performed by the same operator.

### 2.5. Protocol Using the nt-VAL-Jig

**Figure 3:**
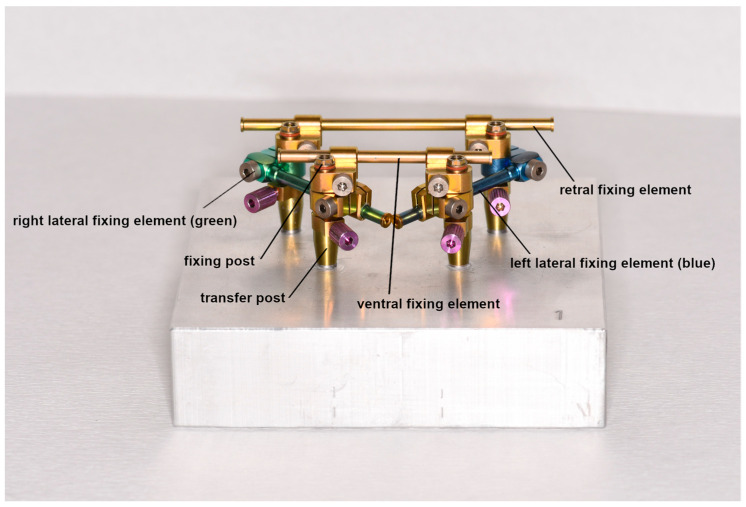
nt-VAL-Jig screwed onto the master model no. 1.

The nt-VAL-Jig was screwed onto the master models according to the manufacturer’s instruction (Figure 3). After the selection of the correct transfer posts (straight or angled) for the used implant system and implant angulation, the transfer posts were placed in the implant analogs, screwed in and hand-tightened. The angled transfer post was used to compensate for the implant divergences to such an extent that the nt-VAL-Jig could be removed. The fixing posts with the knurled screw aligned ventrally and a geometric connection were also hand-tightened to the transfer posts. The left lateral fixing element (blue) and the right lateral fixing element (green) were then pushed onto the fixing posts with the clamping screw aligned ventrally. Additionally, with the clamping screw in a ventral alignment, the long retral fixing element (yellow) and the short ventral fixing element were slid over the lateral fixing elements onto the fixing posts. All screws were fixed with a torque of approx. 10 Ncm to avoid excessive tension. In this position, the nt-VAL-Jig was scanned with Arctica AutoScan. Afterwards, the knurled screws of the fixing posts were unscrewed and the nt-Val-Jig was removed from the transfer posts.

### 2.6. Measurement Protocol

**Figure 4:**
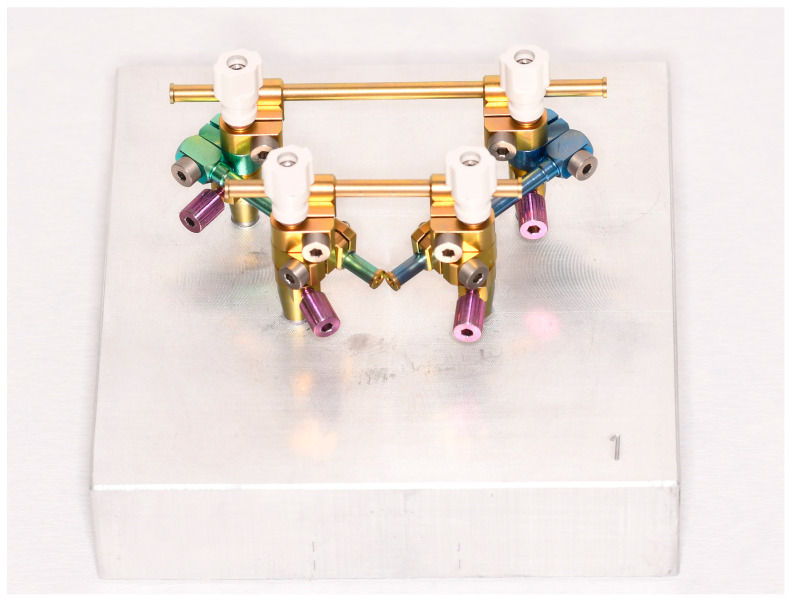
nt-VAL-Jig with scan bodies screwed onto the master model no. 1.

For the measurement, all casts and situations were scanned and digitized with a laboratory scanner (Arctica AutoScan and Dental Teacher; KaVo, Biberach, Germany). The scan bodies (CAM 9.S3D3.800; nt-trading) were hand-screwed onto the implant analogs of the master model first to digitize the master models. After scanning the master models, the scan bodies were placed and hand-screwed in the same alignment on the corresponding stone cast for digitalizing. The nt-VAL-Jig was mounted onto each master model. Screwed onto the master model, the nt-VAL-Jig was equipped with scan bodies on top of the fixing posts and scanned (Figure 4). The nt-VAL-Jig was removed from the transfer posts and digitized without the master model. To verify inaccuracies due to the screwing and to detect whether there could be deviations in different directions leading to exaggerated estimations of the inaccuracy of the impression or if the deviations would be masked in a similar direction, the transfer posts were left on the master model. After removing and scanning the nt-VAL-Jig, the fixing posts with scan bodies were repositioned and screwed onto the master model and scanned. The transfer and fixing posts were screwed onto the corresponding stone casts in the same alignment and were also scanned.

As a result, we were able to conduct three comparisons. Comparison group 1: master model versus stone cast; comparison group 2: master model with screwed transfer and fixing posts versus stone cast with screwed transfer and fixing posts; and comparison group 3: nt-VAL-Jig screwed onto master model versus nt-VAL-Jig removed from master model (Table 2). Ten measurements were performed in each group, one for each master model situation. The same operator performed all the measurements.

### 2.7. Comparison of Implant Position

For the comparison between the open-tray implant level impression and the nt-VAL-Jig, the scan data were imported into GOM-Inspect 2019 Hotfix 6 (GOM, Braunschweig, Germany). With this software, the surface of the scan bodies in every situation and group was superimposed with a three-point prealignment, followed by a best fit registration. The detected point and surface deviations were noted. To determine the point deviation, GOM-Inspect evaluated the Euclidean distance between the nearest points on the two aligned scans and calculated the average. The surface deviation was calculated by determining the (signed) distance between the surface elements of the two matched scans.

In addition to point and surface deviations, false-color images were used to visualize and detect local differences.

### 2.8. Evaluation of Measurements

**Figure 5:**
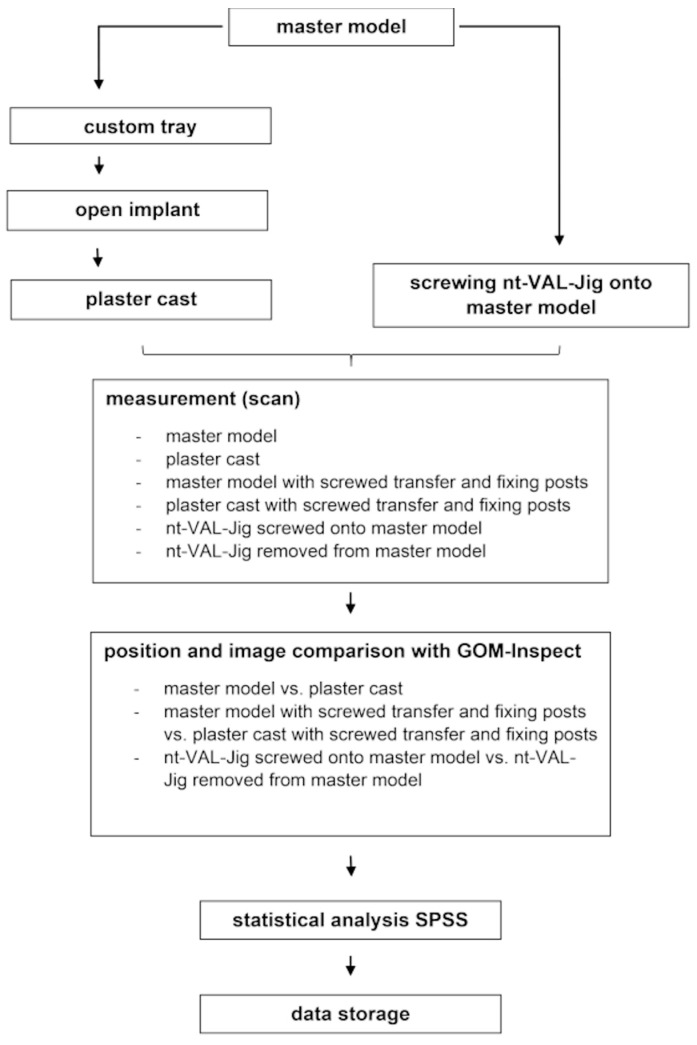
Summary of study protocol.

The point and surface deviations were analyzed with SPSS 27 (IBM, Armonk, NY, USA). Averages and standard deviations were determined both for (absolute) point distances and (signed) face distances of surfaces. Differences between the groups were determined with an analysis of variance (ANOVA) followed by a paired t-test. If a normal distribution and homogeneity of variance could not be assumed, the Kruskal–Wallis test and the Wilcoxon signed-rank test were used. The level of significance was set to α = 0.05. A summary of the protocol is shown in Figure 5.

## 3. Results

**Figure 6:**
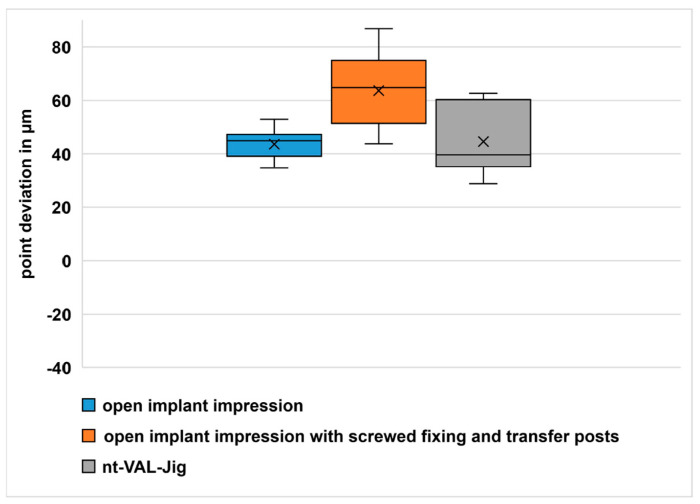
Boxplot diagram point deviation.

**Figure 7:**
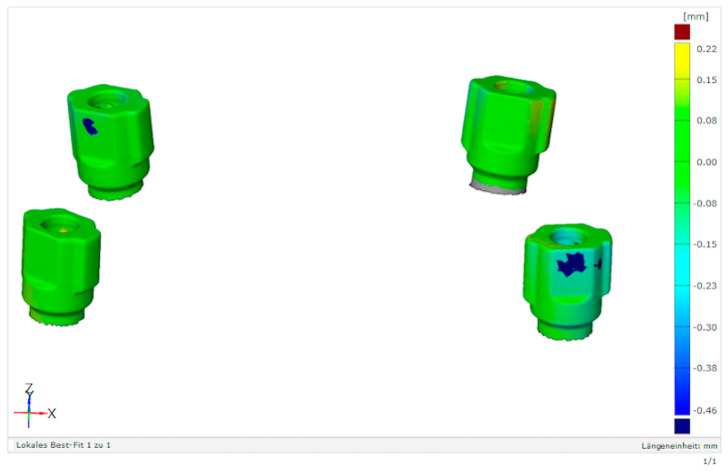
Surface comparison of comparison group 3 with local differences cast no. 6 (local differences in blue color).

The results of the point comparison are presented in Figure 6. The mean point deviation in comparison group one was 43.6 µm, in comparison group two was 63.6 µm and in comparison group three was 44.6 µm. A Kruskal–Wallis test was used for analyzing the differences. A significant difference (*p* = 0.003) between the groups was found. Significant differences between comparison groups one and two and between comparison groups two and three (both *p* < 0.05) were detected with the paired Wilcoxon tests (Table 3). There was no significant difference (*p* > 0.05) between comparison groups one and three. Both groups showed a deviation of approximately 44 µm. There were locally confined differences in the transfer of the implant position in comparison groups two and three (Table 4 and Figure 7) in the surface comparison.

**Figure 8:**
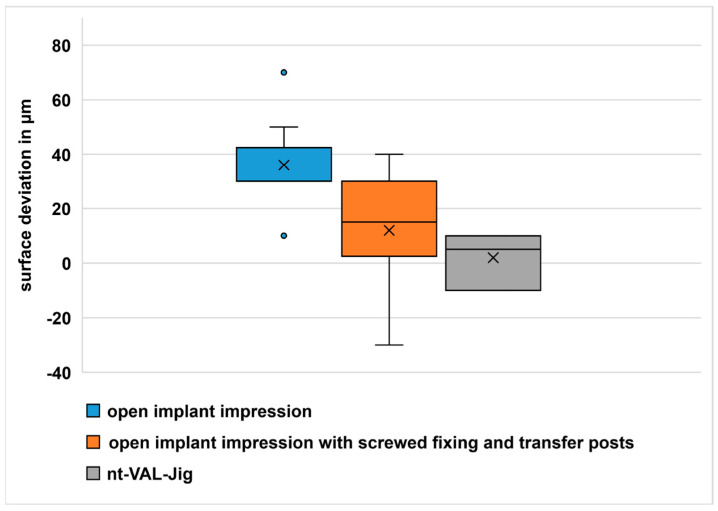
Boxplot diagram surface deviation.

The results of the surface comparison are presented in Figure 8. The mean surface deviation in comparison group one was 36 µm, that of comparison group two was 12 µm and that of comparison group three was 2 µm. The analysis of variance (ANOVA) showed significant differences between the groups (*p* = 0.000). The paired Wilcoxon test (Table 5) showed significant differences between comparison groups one and three and between comparison groups one and two (both *p* < 0.05).

## 4. Discussion

The present study showed no significant difference in the accuracy of localization between a standardized open-tray implant level impression and the nt-VAL-Jig based on a point distance comparison. A larger variation in values was found in the nt-VAL-Jig technique. This could indicate a higher reproducibility of results with the open implant impression. The mean deviation of 43.6 µm was an acceptable difference between the master cast and stone cast and was procedurally unavoidable [18,19]. Assuming the same measured variables, our study did not confirm the claim that the nt-VAL-Jig would achieve better accuracy than the open-tray implant level impression. Furthermore, the effects of the screwing of the posts, such as torque, rotation within the limits of a clearance fit and tension, could explain the differences between the measurements of the open-tray implant level impression and the open implant impression with a screwed transfer and fixing posts. Each screwing could achieve inaccuracies and local differences that were detectable in the surface comparisons. However, the fixing elements of the nt-VAL-Jig seemed to reduce or eliminate these inaccuracies. Local differences were detected mostly on the distal surface of the scan bodies. There may have been contaminations or scanning errors. Otherwise, the color transition in the surface comparison would have been smoother. Another conclusive explanation could not be determined. The implant angulation seemed to have no influence on the transfer of the implant position whether using the open implant impression or the nt-VAL-Jig. In contrast some studies showed an influence of implant angulation on the accuracy of the position transfer [14,26,31], although angulations of 15 degrees seemed to have no effect on the accuracy [28,29,30]. Except for two master casts (number 9 and 10; see Table 1), there were only casts with implant angulations of less than 15 degrees. This could be an explanation for the minimized inaccuracies caused by the implant angulations. The casts with greater angulations than 15 degrees showed no greater inaccuracies either. Maybe this could be explained by the small number of casts and impressions; therefore, that might be a starting point for further investigations.

Furthermore, the test implant system was a flat-to-flat one and, hence, could not be translated to implants with a conical connection. It seemed to be useful to investigate the accuracy of the nt-VAL-Jig using different implant connection types or systems.

In contrast, the evaluation of the surface distances showed a significant difference in the transfer accuracy between the open-tray implant level impression and the nt-VAL-Jig with a higher accuracy for the latter. These diverging results could be explained with the different calculation procedures. The mean point distance was based on the absolute values of Euclidean distances. Therefore, GOM-Inspect detected the closest neighboring points on the superimposed surfaces and determined the distances from this as the absolute value. In contrast, the surface comparison considered the surface normal, i.e., whether one surface was in front of or behind another. Thus, both positive and negative values could contribute to the calculated mean surface distance and partially cancel each other out. As a result, the two measures provided different information about the deviations in the examined bodies, with the latter being less sensitive in certain cases. Thus, the surface comparison should only be used in addition to the point comparison. Alternatively, the positive and negative deviations had to be calculated separately or as the root mean square. However, this option could not be performed in GOM-Inspect. Moreover, this study was limited due to using the point or surface deviation because the implant position and angulation were not detected on the implant level, so that they could be examined approximately. It would have been possible to reconstruct the position of the implants in group two (master model with screwed transfer and fixing posts versus stone cast with screwed transfer and fixing posts) and group three (nt-VAL-Jig screwed onto master model versus nt-VAL-Jig removed from master model) with a calculation from the scan body position. However, as the implant position was only accessible at the top in the master models and plaster casts, and this would have been insufficient for an exact measurement in our opinion, the measurement was performed the way described. In addition, it had to be limited that the open implant impression was created using 3D-printed individual impression trays, and that this may have limited the impression accuracy. However, this method was chosen in order to obtain a uniform thickness of impression material around the impression copings.

Regardless of this fact, the handling of the nt-VAL-Jig turned out to be difficult if the implants were angulated. Several attempts were necessary to choose the correct transfer post and to screw them to fasten the fixing elements. Furthermore, the screws of the fixing elements were easily accessible on the master model, but access could be challenging in clinical use in combination with difficult handling on angulated implants. Training for clinicians and experience in using the device seem necessary. Nevertheless, the nt-VAL-Jig could be used for cross-checking the open-tray implant level impression. Both methods allowed for the transfer of the implant position with good accuracy; therefore, a clinical application of the nt-VAL-Jig seems possible and could be an approach for further investigations. Additionally, it seemed to be possible to use nt-VAL-Jig to correct single implant positions if an implant was placed deeply under the oral mucosa and the impression posts could, therefore, not be fixed tightly in the impression material, e.g., in maxillofacial prosthodontics.

## 5. Conclusions

Within the limits of this in vitro study, it could be concluded that the nt-VAL-Jig allowed for the detection of the implant position with the same accuracy as the open-tray implant level impression. There was no statistically significant difference found between the detection of the implant position through the open-tray implant level impression and the nt-VAL-Jig (*p* > 0.05) in the point comparison, which was more sensitive than the surface comparison. However, in addition to its use for implant localization, the nt-VAL-Jig may also be used to correct faulty implant positions in already produced stone casts.

## Figures and Tables

**Table 1:** Angle and tilt of Camlog implant analogs in the aluminum master casts.

	I_1_		I_2_		I_3_		I_4_	
Cast	Angle *	Tilt **	Angle *	Tilt **	Angle *	Tilt **	Angle *	Tilt **
1	90°	0°	90°	0°	90°	0°	90°	0°
2	100°	10° to o	90°	0°	90°	0°	100°	10° to o
3	80°	10° to v	90°	0°	90°	0°	80°	10° to v
4	80°	10° to d	90°	0°	90°	0°	80°	10° to d
5	100°	10° to m	90°	0°	90°	0°	100°	10° to m
6	90°	0°	100°	10° to o	100°	10° to o	90°	0°
7	90°	0°	80°	10° to v	80°	10° to v	90°	0°
8	90°	0°	80°	10° to d	80°	10° to d	90°	0°
9	90°	0°	60°	30° to d	60°	30° to d	90°	0°
10	115°	25° to o	90°	0°	90°	0°	115°	25° to o

o—oral; v—vestibular; d—distal; m—mesial; * angle to horizontal line; ** tilt in direction.

**Table 2:** Comparison groups.

	aluminum master model	aluminum master model with screwed transfer and fixing posts	nt-VAL-Jig screwed on master model
stone model	comparison group 1 (open-tray implant level impression)	---	---
stone model with screwed transfer and fixing posts	---	comparison group 2 (open-tray implant level impression with screwed transfer and fixing posts)	---
nt-VAL-Jig removed from master model	---	---	comparison group 3 (nt-VAL-Jig)

**Table 3:** Post hoc test point comparison.

	open implant impression	open implant impression with screwed fixing and transfer posts	nt-VAL-Jig
open implant impression	---	0.005 *	0.959
open implant impression with screwed fixing and transfer posts	0.005 *	---	0.009 *
nt-VAL-Jig	0.959	0.009 *	---

* *p* < 0.05—statistically significant.

**Table 4:** Local differences in surface comparison.

Comparison Group	Cast	Implant Analog	Direction	Local Difference (mm)
1	---	---	---	No local differences
2	2	I_1_	Distal	1.00
	5	I_4_	Distal	1.00
	7	I_1_ + I_3_	Distal	0.60
	8	I_1_ + I_3_	Distal	0.60
	9	I_1_	Distal	0.60
	10	I_4_	Distal	1.00
3	2	I_1_	Distal	0.73
	6	I_1_ + I_3_	Distal	≥0.46
	9	I_1_ + I_2_	Distal	≥0.73

Group 1—open-tray implant level impression; group 2—open-tray implant level impression with screwed transfer and fixing posts; group 3—nt-VAL-Jig.

**Table 5:** Post hoc test surface comparison.

	open implant impression	open implant impression with screwed fixing and transfer posts	nt-VAL-Jig
open implant impression	---	0.018 *	0.005 *
open implant impression with screwed fixing and transfer posts	0.018 *	---	0.210
nt-VAL-Jig	0.005 *	0.210	---

* *p* < 0.05—statistically significant.

## Data Availability

All data from this study were presented within Section 3 or are available from the corresponding authors upon reasonable request.
